# The Versatile Effects of Dihydromyricetin in Health

**DOI:** 10.1155/2017/1053617

**Published:** 2017-08-30

**Authors:** Hongliang Li, Qisheng Li, Zhaowen Liu, Kai Yang, Zhixi Chen, Qilai Cheng, Longhuo Wu

**Affiliations:** ^1^College of Pharmacy, Gannan Medical University, Ganzhou 341000, China; ^2^Jiangxi Health Vocational College, Nanchang 330052, China

## Abstract

Dihydromyricetin is a flavonoid isolated from* Ampelopsis grossedentata*, which is traditionally used in China. Dihydromyricetin exhibits health-benefiting activities with minimum adverse effects. Dihydromyricetin has been demonstrated to show antioxidative, anti-inflammatory, anticancer, antimicrobial, cell death-mediating, and lipid and glucose metabolism-regulatory activities. Dihydromyricetin may scavenge ROS to protect against oxidative stress or potentiate ROS generation to counteract cancer cells selectively without any effects on normal cells. However, the low bioavailability of dihydromyricetin limits its potential applications. Recent research has gained positive and promising data. This review will discuss the versatile effects and clinical prospective of dihydromyricetin.

## 1. Introduction

Dihydromyricetin, also known as ampelopsin belonging to flavonoid family, is isolated from* Ampelopsis grossedentata*, which grows widely in the south of China. Traditionally,* Ampelopsis grossedentata* is used as tea in Yao people in China to treat pyretic fever and cough, pain in pharynx and larynx, and jaundice hepatitis. It is also used in nephritis, hepatitis, halitosis, and polyorexia prevention and treatment [1]. Dihydromyricetin is the richest component found in* Ampelopsis grossedentata. *Biologically, recent studies have demonstrated that dihydromyricetin shows multiple health-benefiting activities, including antioxidative, anti-inflammatory, anticancer, antimicrobial, cell death-mediating, and lipid and glucose-metabolism-regulatory activities. In this review article, these biological activities will be discussed comprehensively.

## 2. Chemical Characteristics of Dihydromyricetin

Structurally, due to the highly hydrophilic character, dihydromyricetin shows poor bioavailability and significantly limits its potential medicinal applications. The solubility of dihydromyricetin may be enhanced with temperature increasing from 0.2 mg/ml at 25°C to 0.9 mg/ml at 37°C. The diagrams of phase-solubility show that dihydromyricetin solubility is positively correlated with the concentration of hydroxypropyl-*β*-cyclodextrin, PVP K30, and PEG6000. Thus, the solubility of dihydromyricetin increases to 2.8 mg/ml at 25°C and 9.6 mg/ml at 37°C, respectively [2]. In addition, enzyme-acylated product of dihydromyricetin improves its lipid-solubility and also exhibits a good antioxidative activity [3]. The poor bioavailability is further supported by the pharmacokinetic characteristics, which show *C*_max_ (21.63 ± 3.62 ng/mL) and* t*_1/2_ (3.70 ± 0.99 h) after oral administration [4]. Similar results are obtained but show different pharmacokinetic characteristics of dextroisomer and racemate in dihydromyricetin: *C*_max_ (81.3 and 107 ng/mL), AUC_0-∞_ (42.8 and 32.0 mg × min/L), and* t*_1/2_ (288 and 367 min), respectively [5].

**Figure 1:**
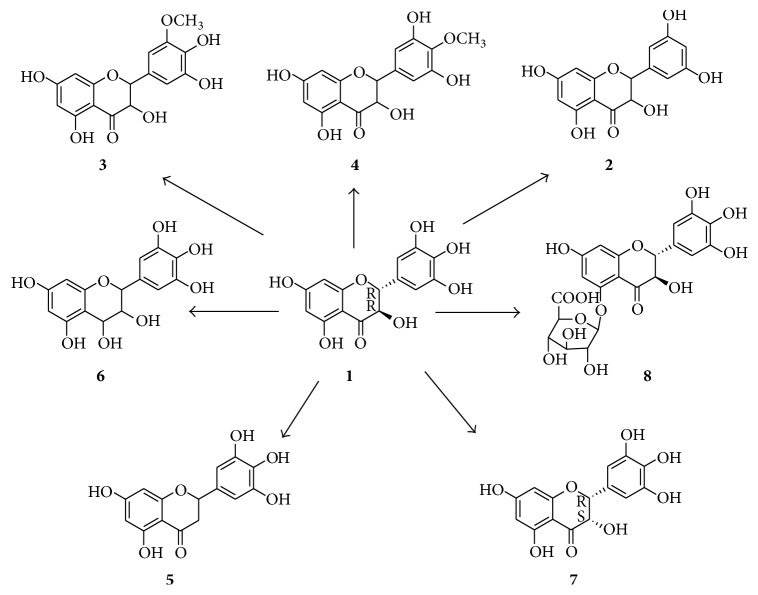
The structure of dihydromyricetin (1) and its metabolites (2–8).

However, the low bioavailability of dihydromyricetin may be also partially attributed to its poor structural stability. Dihydromyricetin decomposes when exposing to light, pH buffer, pepsin, and pancreatin enzymes. Generally, the metabolites of flavonoids are produced by hydrolysis, ring fission, and reduction [6]. Dihydromyricetin can be transformed into seven metabolites in rats [7] (Figure 1). They are 5,7,3′,5′-tetrahydroxyflavanonol (2), 5,7,4′,5′-tetrahydroxy-3′-methoxyflavone (3), 5,7,3′,5′-tetrahydroxy-4′-methoxyflavone (4), 5,7,3′,4′,5′-pentahydroxyflavanone (5), 3,4,5,7,3′,4′,5′-hepthydroxyflavan (6), (2R,3S)-5,7,3′,4′,5′-pentahydroxyflavanonol (7), and dihydromyricetin-O-5-*β*-D-glucuronide (8).

## 3. Oxidative Stress-Mediating Activity

Oxidative stress is a state of cellular homeostasis imbalance, characterized as reactive oxygen species (ROS) production overweighting the antioxidant enzyme system. Excessive ROS contributes to mitochondria-dependent apoptosis. The mechanistic chemistry in radical scavenging ability of dihydromyricetin has been approved in protection against mesenchymal stem cells damage [8]. The antioxidative activity of dihydromyricetin is also confirmed by two model systems, including cooked ground beef and soybean oil [9]. Excessive ROS may acts as a dominant factor contributing to myocardial fibrosis. Cardiac fibroblast may be activated by angiotensin II through induction of ROS production, promoting proliferation, and collagen synthesis. Dihydromyricetin restores these adverse effects induced by angiotensin II, as indicating by decreased levels of ROS and MDA, attenuated expression of p22^phox^ (a subunit of NADPH oxidase), and increased total antioxidant capacity [10]. Similar results are showed in the antioxidative effect of dihydromyricetin on attenuating angiotensin II-induced cardiomyocyte hypertrophy [11].

Excessive ROS is also correlated with neurogenerative diseases. 3-Nitropropionic acid may induce motor dysfunction and learning and memory impairments through hyperactivation of ROS production. Dihydromyricetin significantly restores metabolic abnormality in striatum, improves the expression of antioxidant system, and inhibits mitochondria-dependent apoptosis [12]. Memory impairments are also subject to hypobaric hypoxia, which often induces oxidative stress in the brain. Dihydromyricetin has been showed to suppress ROS production and attenuate lipid peroxidation in the hippocampus, promoting mitochondrial biogenesis and improving mitochondrial functions (Table 1). In addition, dihydromyricetin protects neurons from hypobaric hypoxia through amelioration of Sirt3-FOXO3a signaling-induced oxidative stress [13].

Oxidative stress has been considered as the critical factor correlating with nephrotoxicity induced by cisplatin. In HK-2 cells, dihydromyricetin may protect against such nephrotoxicity through attenuation of oxidative stress and inflammatory stress, leading to inhibition of apoptosis [14]. ROS in osteocytes contributes to osteoporosis formation. In MG63 cells, dihydromyricetin effectively exhibits antioxidative activity to scavenge ROS and leads to attenuation of caspase-3 and caspase-9 and inhibition of cell apoptosis [15]. In HUVECs, dihydromyricetin ameliorates H_2_O_2_-induced oxidative stress against apoptosis mitochondria dependently [16]. In addition, dihydromyricetin may increase the total antioxidant capacity and attenuate ROS generation and NOX2 expression. Thus, dihydromyricetin ameliorates the cytotoxicity induced by oxLDL, as indicated by monocytes adhesion and oxidative stress [17].

P62 has been demonstrated to competitively bind to Keap1, which plays a negative role in modulating Nrf2 activity. The complex p62-Keap1-LC3II promotes Keap1 degradation, which further activates Nrf2 in a positive feedback loop. Dihydromyricetin significantly induces p62 expression and subsequent Nrf2 and HO-1 activation, leading to attenuation of oxidative stress and hepatoprotection against toxicity induced by ethanol [18].

## 4. Anti-Inflammatory Activity

NF-*κ*B signaling has been demonstrated to play a critical role in regulating the expression of target genes relating to inflammation. The subunit I*κ*B*α*, as a negative controller, can be degraded after phosphorylation modification, leading to activation and nuclear translocation of p65 and subsequent promotion of NF-*κ*B target genes expression. The computational docking assays show that dihydromyricetin binds to a novel binding site IKK*β*-Cys46, which plays a pivotal role in the pathogenesis of inflammation. The delayed-type hypersensitivity and an* IKKβ*^*C46A*^ transgenic mouse model confirm that Cys46 is the binding site for dihydromyricetin to be responsible for suppression of NF-*κ*B signaling [55]. In LPS-induced RAW2264.7 microphages, dihydromyricetin attenuates IKK*β* activity and IKK*α*/*β* phosphorylation, leading to inhibiting p65 phosphorylation and nuclear translocation and suppressing target genes expression, including COX-2 and iNOS [19]. Similar results showed that dihydromyricetin inhibits the phosphorylation of NF-*κ*B, p38, and JNK, but not ERK1/2 in LPS-induced RAW2264.7 microphages [20].

Dihydromyricetin has been reported to inhibit TNF-*α*-induced inflammation through inactivation of NF-*κ*B signaling in HeLa cells. Specifically, dihydromyricetin dephosphorylates and inhibits the degradation of I*κ*B*α*, inactivates p65 nuclear translocation and downregulates the TNF-*α*-induced expression of TRAF2 and RIP1. In addition, dihydromyricetin also downregulates the expression of NF-*κ*B target genes, including c-IAP2, Bcl-2, TRAF1, iNOS, cyclin D1, COX-2, ICAM-1, MMP-9, and VEGF [21] (Table 1). In asthmatic mouse model, ovalbumin promotes the secretion of proinflammatory cytokines, IgE, and IgG1 and the infiltration of inflammatory cells into the bronchoalveolar lavage. Dihydromyricetin has been demonstrated to significantly reduce ovalbumin-induced inflammatory activities [22].

## 5. Anticancer Activity

ROS may act as a messenger to balance redox signaling to determine cell fates. Higher ROS production and oxidative stress are positively correlating with carcinogenesis. Interestingly, dihydromyricetin may regulate cell death potentially through mediating ROS generation. Dihydromyricetin, in a dose-dependent manner, promotes ROS generation and activation of mitochondria-dependent apoptosis in human hepatocarcinoma HepG2 cells [23]. Mechanistically, dihydromyricetin triggers mitochondria-dependent apoptotic pathway through downregulating Akt/Bad signaling. More specific, dihydromyricetin inhibits the phosphorylation of Akt-Ser473 and Bad-Ser112/Ser136 and enhances Bax and Bad proteins expression, leading to formation of Bcl-2/Bcl-xL heterodimers and activation of Bax-stimulated mitochondrial apoptosis in HepG2 cells [24]. In mouse hepatocellular carcinoma Hepal-6 cells, dihydromyricetin dose-dependently induces cell apoptosis through downregulation of TGF*β*/Smad3 pathway and NOX4/ROS pathway [27]. In addition, dihydromyricetin significantly inhibits the expression of MMP-9, but not MMP-2, which is the key factor responsible for the migration and invasion of SK-Hep-1 cells. This underlying mechanism of dihydromyricetin in antimetastasis is related to the decreased phosphorylation levels of p38, ERK1/2, and JNK, and the increased expression of PKC-*δ* [28] (Table 1).

In A2780 and SKOV3 cell lines, dihydromyricetin dose- and time-dependently inhibits cellular proliferation and causes cell cycle arrest in G0/G1 and S phases. The activation of p53 signaling and the suppression of survivin expression are involved in dihydromyricetin-induced ovarian cancer cell apoptosis. Survivin, an inhibitors of apoptosis proteins (IAPs) family, is a key factor in cellular chemotherapy-related resistance. Thus, dihydromyricetin may promote the resistant ovarian cancer cells to resensitize to paclitaxel and doxorubicin through suppression of survivin expression [29]. On molecular mechanism of drug resistance in colorectal cancer HCT116/L-OHP cells, dihydromyricetin significantly inhibits the promoter activity and the expression of multidrug resistance protein 2 (MRP2), leading to chemosensitivity of cells to oxaliplatin. In addition, dihydromyricetin also attenuates the nuclear translocation of erythroid 2 p45 related factor 2, a MRP2 regulator [30].

In osteosarcoma, dihydromyricetin may upregulate the expression of p21 and cause cell cycle arrest in G2-M phage, leading to cell apoptosis. The molecular mechanism is associated with dihydromyricetin-induced activation of AMPK*α*-GSK-3*β*-Sox2 signaling pathway [31] (Table 1). In human melanoma SK-MEL-28 cells, dihydromyricetin promotes the expression of p21 and p53 and attenuates the expression of cdc2, p-cdc-2, and cdc25A, causing cell cycle arrest in G1/S phase. In addition, dihydromyricetin activates cell apoptosis through upregulation of the proapoptotic factor Bax and downregulation of NF-*κ*B pathway and p38 pathway [32]. In HepG2 and Hep3B cell lines, dihydromyricetin may cause cell cycle arrest in G2/M phase through activation of Chk1/Chk2/cdc25C. However, deficiency of p53 and Chk1 does not cause dihydromyricetin-induced G2/M arrest [33].

## 6. Cell Death-Mediating Activity

Apoptosis is a process of programmed cell death, which exhibits a critical role in cellular physiopathology of various tissues and organs. Dihydromyricetin, in a dose-dependent manner, downregulates the expression of p53 and upregulation Bcl-2 expression, leading to activation of apoptosis in gastric cancer cell [34]. Interestingly, dihydromyricetin promotes cell apoptosis through reduction of TGF*β* and activation of p53 signaling pathways in HepG2 cells [25]. Consistently, dihydromyricetin downregulates Bcl-2 expression and increases Bax/Bcl-2 ratio through upregulation of p53 signaling pathway in HepG2 cells [56]. Dihydromyricetin exhibits a selective cytotoxicity against non-small-cell lung cancer (NSCLC) cells (A549 and H1975), but not against normal cells (WI-38). This might be related to dihydromyricetin-triggered ROS generation, which causes a mitochondria-dependent apoptosis. In addition, dihydromyricetin promotes ROS-induced ERK1/2 and JNK1/2 signaling pathways, which can be reversed by N-acetylcysteine [35].

Dihydromyricetin can induce not only apoptosis but also autophagy in human melanoma (SK-MEL-28) cells. Dihydromyricetin potentiates ROS generation, which can be counteracted by N-acetyl-L-cysteine (NAC). The mechanism of dihydromyricetin-induced autophagy is related to upregulation of NF-*κ*B phosphorylation induced by ROS [36]. Similarly, dihydromyricetin induces cardiac autophagy and protects against apoptosis in STZ-induced diabetic mice, as indicated by upregulation of Beclin1, Atg7, and Bcl-2 expression and LC3 II/LC3 I ratio and downregulation of p62, caspase-3/-9 levels. Further, dihydromyricetin may promote AMPK and ULK1 phosphorylation, improve mitochondrial functions, and subsequently prevent diabetic cardiomyopathy [37]. mTOR, a master regulator belonging to PI3K related kinase family, regulates the activation of autophagy. mTOR can be phosphorylated and regulated by PI3K/Akt, ERK1/2, and AMPK through regulating TSC2 and TSC1/2 phosphorylation. Dihydromyricetin has been reported to activate AMPK and attenuate the expression of p-ERK1/2 and p-Akt, leading to inhibition of mTOR and activation of autophagy in HepG2 cell lines [26] (Table 1).

AMPK increases the transcriptional activity of FOXO3a through the phosphorylation at Ser588. In liver I/R injury, dihydromyricetin also increases the mRNA expression of autophagy-related genes, such as BECN1, LC3, Atg5, and Atg12, protecting liver cell against apoptosis. This might be associated with upregulation of FOXO3a protein expression, nuclear translocation, and phosphorylation at Ser588 induced by dihydromyricetin [38]. FOXO3a activity is also mediated by its acetylation induced by p300/CBP or Sirt. However, the acetylation levels of FOXO3a are not changed in the cytosol, indicating that FOXO3a acetylation does not play an important role in dihydromyricetin-induced autophagy [38]. In head and neck squamous cell carcinoma (HNSCC), dihydromyricetin promotes the phosphorylation and activation of STAT3 and subsequent induction of autophagy through producing ROS. Specifically, dihydromyricetin induces the upregulation of autophagic markers such as Beclin1, LC3, and p62. In addition, dihydromyricetin also promote HNSCC cells apoptosis [39] (Table 1).

## 7. Metabolism-Mediating Activity

Flavonoids are also partial agonists of PPAR*γ*, which exhibits an inhibitory effect on diabetes. Upregulation of diabetogenic adipokines expression and downregulation of adiponectin expression are mediated by PPAR*γ*-Ser273 phosphorylation, which is regulated by ERK/CDK5 signaling pathway. In Zucker diabetic fatty rats, dihydromyricetin may inhibit the phosphorylation of PPAR*γ*-Ser273 through attenuation of ERK/CDK5 signaling pathway, leading to retardation of hyperglycemia onset and amelioration of insulin resistance without weight gain [40]. Management of insulin resistance in skeletal muscle becomes a strategy for type II diabetes (T2D) treatment. Dihydromyricetin increases skeletal muscle insulin sensitivity, as indicated by upregulation of p-IRS-1 and p-AKT expression, by inducing formation of autophagosomes partially through activation of AMPK-PGC-1*α*-Sirt3 pathway in C2C12 myotubes [41, 42] (Table 1).

In LDL receptor knockout* (LDLr*^−/−^) mice, dihydromyricetin decreases high-fat diet-induced serum levels of ox-LDL, IL-6, and TNF-*α* and increases PPAR*α*, LXR*α*, and ABCA1 expression, leading to amelioration of hyperlipidemia, suppression of hepatic lipid accumulation, and inhibition of foam cell formation and cholesterol efflux [17]. This is supported by the* ApoE*^−/−^ mouse model, which shows that dihydromyricetin can significantly prevent the development of weight gain, hyperlipidemia, and atherosclerosis induced by a Western diet (high cholesterol, high sucrose, and high-fat) [57]. Dyslipidemia constitutes a major health problem in inducing atherosclerosis. Many flavonoids including naringenin, quercetin, and dihydromyricetin are involved in glucose and lipid profiles improvement. Synergized with benzo[a]pyrene (BaP), *β*-naphthoflavone (BNF) activates CYP1A1 expression and CYP1A1-mediated 7-ethoxyresorufin O-deethylation (EROD). Dihydromyricetin has been demonstrated to promote tumorigenesis induced by BaP in small intestine [58]. However, dihydromyricetin alone does not show any significant effects on metabolic activity of CYP1A1/2 and CYP2B1 enzymes [59].

Irisin is a new myokine derived from the fibronectin type III domain-containing protein 5 (FNDC5). PGC-1*α* regulates the expression of FNDC5 mRNA and the metabolism of irisin, which is correlated with body mass index (BMI). Dihydromyricetin has been demonstrated to increase irisin levels in serum and upregulate the FNDC5 expression through partially activating PGC-1*α* pathway, leading to amelioration of metabolic diseases [44]. Palmitate has been identified as a major inducer of insulin resistance in obesity. Also, palmitate can downregulate the expression of slow-twitch fiber proportion, AMPK, and PGC-1*α* and upregulate the expression of folliculin-interacting protein 1 (FNIP1) and folliculin in C2C12 myotubes. These effects induced by palmitate could be abrogated by dihydromyricetin administration [43].

## 8. Neuroprotective Activity

MicroRNAs (miRs) have been demonstrated to be involved in the development of Alzheimer's disease (AD). Sirt, a direct substrate of miR-34a, can promote cell tolerance to aging through induction of autophagy. In aging models, dihydromyricetin downregulates the D-gal-induced expression of miR-34a and p53/p21 pathways and upregulates Sirt1 expression. mTOR negatively modulates autophagy activation. Dihydromyricetin may increase the phosphorylation of mTOR at Ser2448 and inactivate it in D-gal-induced models, leading to activation of autophagy [45]. In Parkinson's disease (PD), dihydromyricetin also exhibits neuroprotective activity in behavioral tests through attenuation of MPTP-induced cytotoxicity, ROS generation, and GSK-3*β* activation dose- and time-dependently [46] (Table 1). L-Dopa has been implicated in PD management. Catechol O-methyltransferase (COMT) may decrease the bioavailability of L-dopa. Dihydromyricetin has been demonstrated to benefit PD management through inhibition of COMT activity dose-dependently [60].

Dihydromyricetin is also the main component of Hovenia, which is traditionally used for treatment of alcohol hangovers. It has been demonstrated that dihydromyricetin may exhibit the protective effects against alcohol intoxication and alcohol tolerance. The molecular mechanism might be associated with competitively binding of dihydromyricetin to BZ sites on GABAARs [61]. Fetal alcohol exposure (FAE) promotes long-lasting alternations in behavior and physiology, which might be related to dysfunction of GABAARs in hippocampi. In rat models, dihydromyricetin effectively prevents FAE disorders through regulation of GABAARs [62]. Dysfunction of GABAARs in neurotransmission also contributes to AD development. In transgenic (TG2576) and Swedish transgenic (TG-SwDI) mice, dihydromyricetin may reduce A*β* peptide production and restore gephyrin levels, GABAergic transmission, and functional synapses, leading to improvement of clinical symptoms [63].

## 9. Miscellaneous Section

Dihydromyricetin also exhibits anti-bacterial activity against* Staphylococcus aureus*. The possible mechanism is that dihydromyricetin may disrupt the integrity and the fluidity of membrane. In addition, dihydromyricetin also binds to intracellular DNA through the groove-binding mode in* S. aureus *[64]. This is inconsistent with reports from Huang et al. (2015) that dihydromyricetin does not significantly inhibit* S. aureus* PriA, which is an important helicase for DNA replication restart [65]. Dihydropyrimidinase, a key member in the chain of pyrimidine catabolism, plays an important role in metabolism of DNA base in* Pseudomonas aeruginosa *PAO1. Abrogation of dihydropyrimidinase may lead to inhibition of bacterial growth and promotion of death. Dihydromyricetin substrate-dependently docks into the active site of dihydropyrimidinase and inhibits its activity with IC_50_ value of 80 *μ*M [66].

Dihydromyricetin decreases the expression of MDA, blood urea nitrogen, and kidney tissue molecule-1 and inhibits cell apoptosis, protecting against kidney injury induced by LPS. [47]. On protection against acute liver injury, dihydromyricetin exhibits anti-inflammatory, antiapoptotic, and proliferation-accelerating activities in carbon tetrachloride- (CCl_4_-) induced hepatocytes through upregulation of JNK expression [48]. Melanogenesis is positively regulated by MAPK pathway, cAMP/PKA pathway, and PKC pathway through upregulating of CREB/MITF axis. Dihydromyricetin has been demonstrated to attenuate the activities of these three signaling pathways and inhibits the expression of CREB and MITF, leading to blockage of melanogenesis in B16F10 melanoma cells [49] (Table 1). Wnt/*β*-catenin signaling pathway plays a pivotal role in mediating osteogenic differentiation in bone mesenchymal stem cells (BMSCs). Evidences show that dihydromyricetin decreases the expression of kickkopf-1 and sclerostin and increase *β*-catenin transcriptional activity, resulting in enhancing osteogenic differentiation in vitro [67].

## 10. Clinical Prospective

It is well proved that high-fat diet may severely cause hyperlipidemia, hepatic steatosis, and type II diabetes. In high-fat diet rats, dihydromyricetin improves glucose uptake, promotes glucose transporter 1 (GLU1) translocation, and enhances Krebs cycle activity, leading to amelioration of insulin resistance. Specifically, dihydromyricetin reverses the decreased levels of CS, SDHA, and DLST induced by high-fat diet. Similarly, the increased levels of serine, leucine, asparagine, SSA, 5-L-glutamyl-alanine, and L-methylhistidine are also restored by dihydromyricetin. These are associated with downregulation of phosphorylation of IRS-Ser612 and upregulation of Akt and AMPK, resulting in inhibitory phosphorylation of GSK-3*β* and reduction of G6Pase and PEPCK expression [50] (Table 1). Nonalcoholic fatty liver disease is characterized by accumulation of TG and TC in the cytoplasm of hepatocytes. Dihydromyricetin exhibits inhibitory effects on this accumulation and ROS generation, which are related to regulation of AMPK, AKT, and PPAR*γ* pathways in oleic acid-induced L02 and HepG2 cells [51]. In a double-blind clinical trial, either two dihydromyricetin or two placebo capsules are applied for twice daily and three months. Dihydromyricetin supplementation may significantly ameliorate the serum levels of glucose, LDL-C, GGT, alanine, AST, and Apo B, resulting in dihydromyricetin-enhanced metabolism of glucose and lipid. In addition, dihydromyricetin also downregulates the expression of TNF-*α*, CK-18 fragment, and FGF21 [52].

Oxidative stress may exaggerate ischemia and reperfusion (I/R) injury, leading to cell apoptosis. In rats* in vivo* and H9c2 cardiomyocytes* in vitro*, dihydromyricetin provides effective protection against I/R-induced injury through activation of PI3K/Akt and HIF1*α* signaling pathways, leading to augment of cellular antioxidant capacity and inhibition of apoptosis. These are characterized by upregulation of antiapoptotic factors Bcl-2 and Bcl-XL and downregulation of proapoptotic factors Bax, Bnip3, cleaved caspase-3/-9, and cytochrome* c* [53]. Methylglyoxal (MG), an endogenous toxic compound from the glycolytic pathway, may accumulate and impair cognitive dysfunction in metabolic diseases. MG may potentiate oxidative stress and calcium overload, leading to activation of mitochondrial apoptosis in PC12 cells. This might be associated with impairing of BLUT4 translocation and downregulating the expression of glyoxalase 1 and p-AMPK*α*. Dihydromyricetin exhibits a protective role in treating diabetic encephalopathy through ameliorating MG toxicity [54].

Combined with nedaplatin, dihydromyricetin synergistically induces apoptosis in p53/Bcl-2 signaling-dependent manner in hepatocellular carcinoma (SMMC7721 and QGY7701) cells. In addition, dihydromyricetin selectively protects normal hepatocytes (HL7702) against damage induced by nedaplatin [68]. Similarly, dihydromyricetin has been reported to show no cytotoxicity to normal hepatocytes but significant inhibition of cellular proliferation and activation of apoptosis in a p53-dependent manner in HCC cells [69]. Dihydromyricetin selectively induces tumor cells mitochondrial apoptosis and synergistically potentiates the cytotoxicity of cisplatin in HepG2 and SMMC-7721. This is possibly related to dihydromyricetin-induced enhancement of p53 phosphorylation at Ser15 [70]. Adriamycin causes serious cardiotoxicity, as indicated by increased levels of ALT, LDH, and CKMB in the serum, leading to activation of apoptosis. Dihydromyricetin exhibits the cardioprotective activity that it ameliorates adriamycin-induced cardiotoxicity and synergistically potentiates anticancer activity of adriamycin p53-dependently [71].

## 11. Concluding Marks

In this review, we focus on dihydromyricetin biological activities, including antioxidative, anti-inflammatory, anticancer, and lipid and glucose metabolism-regulatory activities. ROS acts as a dual role in redox signaling to control cell fates. Dihydromyricetin may scavenge ROS to ameliorate oxidative stress for protection or potentiate ROS generation to kill cancer cells. Intriguingly, dihydromyricetin improves metabolic diseases through mediating lipid and glucose metabolism. These make dihydromyricetin to be a possible candidate for clinical potentials. However, the low bioavailability of dihydromyricetin limits its applications. Thus, more efforts should be needed for its underlying mechanism in biology.

## Figures and Tables

**Table 1:** The biological activities of dihydromyricetin.

Cell types/animals	Biological activities	Ref
Mesenchymal stem cells	Radical scavenging↑	[8]
Cardiac fibroblast	ROS↓; MDA↓; p22^phox^↓; SOD↑; thioredoxin↑; total anti-oxidant capacity↑; proliferation↓; collagen synthesis↓	[10]
Cardiomyocyte	ROS↓; MDA↓; NO↓; p22^phox^↓; SOD↑; total anti-oxidant capacity↑; cGMP↑; hypertrophy↓	[11]
Rats	motor dysfunction↓; learning and memory impairments↓; MDA↓; glucose metabolism↑; SOD↑; Bcl-2↑; Bax↓; Cleaved Caspase-3↓	[12]
Rats and HT-22 cells	PGC-1*α*↑; TFAM↑; SIRT3↑; FOXO3 deacetylation↑; ROS↓; synaptic plasticity↑; oxidative stress↓; mitochondrial function↑	[13]
HK-2 cells	MDA↓; CAT↑; SOD↑; Bcl-2↑; IL-1*β*↓; IL-6↓; TNF-*α*↓; MCP-1↓	[14]
MG63 cells	ROS↓; caspase-3↓; caspase-9↓	[15]
HUVECs	ROS↓; p53↓; Bcl-2↑; Bax↓; caspase-3↓; caspase-9↓; PARP↓	[16]
	ox-LDL↓; IL-6↓; TNF-*α*↓; PPAR*α*↑; LXR*α*↑; ABCA1↑; lipid accumulation↓; ROS↓; NO2↓	[17]
C57BL/6 mice	Nrf2/HO-1↑; CYP2E1↓; p62↑; Keap1↓; LC3-II↑; Beclin 1↑	[18]
RAW2264.7	IKK*β*↓; p-IKK*α*/*β*↓; p-p65↓; COX-2↓; iNOS↓	[19]
	p-NF-*κ*B↓; p-p38↓; p-JNK↓	[20]
HeLa cells	p-I*κ*B*α*↓; p-p65↓; TRAF2↓; RIP1↓; c-IAP2↓; Bcl-2↓; TRAF1↓; iNOS↓; cyclin D1↓; COX-2↓; ICAM-1↓; MMP-9↓; VEGF↓	[21]
Mouse	IL-4↓; IL-5↓; IL-13↓; IgE↓; IgG1↓	[22]
HepG2 cells	ROS↓; GSH↓; ATP↓; caspase-9↑; caspase-8↑; caspase-3↑; HO-1↑; BAK↑; Bcl-2↓	[23]
	p-Akt-Ser473↓; p-Bad-Ser112/Ser136↓; Bax↑; Bad↑	[24]
	TGF*β*↓; p53↑; Bcl-2↓	[25]
	AMPK↑; p-ERK1/2↓; p-Akt↓; mTOR↓; autophagy↑	[26]
Hepal-6 cells	TGF*β*/Smad3↓; NOX4/ROS↓	[27]
SK-Hep-1 cells	MMP-9↓; p-p38↓; p-ERK1/2↓; p-JNK↓; PKC-*δ*↑	[28]
A2780; SKOV3	p53↑; survivin↓	[29]
HCT116/L-OHP	MRP2↓; erythroid 2 p45 related factor 2↓	[30]
Osteosarcoma	p21↑; AMPK*α*-GSK-3*β*-Sox2↑	[31]
SK-MEL-28 cells	p21↑; p53↑; cdc2↓; p-cdc-2↓; cdc25A↓; Bax↑; IKK*α*↓; p65↓; p-p38↓	[32]
HepG2; Hep3B	Chk1/Chk2/cdc25C↑	[33]
Gastric cancer cell	p53↓; Bcl-2↑	[34]
NSCLC	ROS↑; ERK1/2↑; JNK1/2↑	[35]
SK-MEL-28	ROS↑; p-NF-*κ*B↑	[36]
Diabetic mice	Beclin1↑; Atg7↑; Bcl-2↑; LC3 II/LC3 I↑; p62↓; caspase-3/-9↓; p-AMPK↑; p-ULK1↑	[37]
Liver I/R injury	BECN1↑; LC3↑; Atg5↑; Atg12↑; FOXO3a↑	[38]
HNSCC	ROS↑; STAT3↑; Beclin1↑; LC3↑; p62↑	[39]
Diabetic fatty rats	p-PPAR*γ*-Ser273↓; ERK/CDK5↓; insulin resistance↓	[40]
C2C12 myotubes	p-IRS-1↑; p-AKT↑; AMPK-PGC-1*α*-Sirt3↑; autophagosomes↑	[41, 42]
	AMPK↑; PGC-1*α*↑; FNIP1↓	[43]
*LDLr* ^−/−^ mice	ox-LDL↓; IL-6↓; TNF-*α*↓; PPAR*α*↑; LXR*α*↑; ABCA1↑; hyperlipidemia↓; foam cell↓; cholesterol efflux↓	[17]
Rats; humans	irisin↑; FNDC5↑; PGC-1*α*↑	[44]
AD rats	miR-34a↓; p53/p21↓; Sirt1↑; p-mTOR-Ser2448↑; autophagy↑	[45]
PD rats	ROS↓; GSK-3*β*↓	[46]
LPS-induced rat kidney	blood urea nitrogen↓; molecule-1↓; MDA↓; apoptosis↓	[47]
Rat liver	JNK↑; inflammation↓; apoptosis↓; proliferation↑	[48]
B16F10	MAPK↓; cAMP/PKA↓; PKC↓; CREB↓; MITF↓	[49]
Rats	glucose uptake↑; GLU1↑; Krebs↑; insulin resistance↓; CS↑; SDHA↑; DLST↑; serine↓; leucine↓; asparagine↓; SSA↓; 5-L-glutamyl-alanine↓; L-methylhistidine↓; p-IRS-Ser612↓; Akt↑; AMPK↑; GSK-3*β*↓; G6Pase↓; PEPCK↓	[50]
L02; HepG2 cells	TG↓; TC↓; ROS↓; AMPK↑; AKT↑; PPAR*γ*↑	[51]
Humans	glucose↓; LDL-C↓; GGT↓; alanine↓; AST↓; Apo B↓; TNF-*α*↓; CK-18 fragment↓; FGF21↓	[52]
Rats; H9c2	PI3K/Akt↑; HIF1*α*↑; Bcl-2↑; Bcl-XL↑; Bax↓; Bnip3↓; cleaved caspase-3/-9↓; cytochrome *c*↓	[53]
PC12 cells	oxidative stress↓; calcium overload↓; p-AMPK*α*↑	[54]
